# Suppressive Effect of Quercetin on Nitric Oxide Production from Nasal Epithelial Cells* In Vitro*

**DOI:** 10.1155/2018/6097625

**Published:** 2018-07-05

**Authors:** Nachi Ebihara, Kana Takahashi, Haruka Takemura, Yuko Akanuma, Kazuhito Asano, Masataka Sunagawa

**Affiliations:** ^1^Department of Physiology, School of Medicine, Showa University, Tokyo, Japan; ^2^Division of Physiology, School of Nursing and Rehabilitation Sciences, Showa University, Yokohama, Japan

## Abstract

Nitric oxide (NO) is known to play pivotal roles as one of the final effector molecules in the development of allergic diseases, including allergic rhinitis (AR). Although quercetin has been reported to attenuate the clinical conditions of AR, its influence on NO production is not well defined. The present study aimed to examine the influence of quercetin on* in vitro* NO production from nasal epithelial cells after interleukin- (IL-) 4 stimulation. Human nasal epithelial cells (HNEpCs) at a concentration of 1 x 10^5^ cells/ml were stimulated with 10.0 ng/ml of IL-4 in the presence and absence of quercetin. After 48 hours, the culture supernatants were collected and assayed for NO (NO_2_ and NO_3_) using the Griess method. The influences of quercetin on the transcription factor, STAT6, activation, and iNOS mRNA expression were also examined using ELISA and real-time quantitative RT-PCR, respectively. Addition of quercetin to cell cultures caused suppression of NO production from HNEpCs after IL-4 stimulation. The minimum concentration of quercetin that caused significant suppression was 1.0 nM. Treatment of cells with quercetin at more than 1.0 nM suppressed STAT6 activation and iNOS mRNA expression induced by IL-4 stimulation. The present results strongly suggested that quercetin favorably modified the clinical condition of AR through the suppression of NO production from nasal epithelial cells after IL-4 stimulation.

## 1. Introduction

Allergic rhinitis (AR) is a chronic inflammatory condition of the nasal mucosa in sensitized patients and is mediated by IgE-dependent immune responses to several types of environmental allergens, such as pollen, house dust, and animal dander. After inhalation, these allergens are internalized and processed by antigen presenting cells, especially dendritic cells and macrophages, and the peptide fragments of these allergens are presented to Th2-type helper T cells [1, 2]. Activated Th2-type helper T cells secrete interleukin- (IL-) 4 and IL-13, which trigger the production of antigen-specific IgE from B cells. The produced antigen-specific IgE binds to high-affinity receptors for IgE (Fc*ε*R) that are expressed on the surface of mast cells. When the specific allergen is reinhaled into the nose, the adjacent IgE molecules cross, and mast cell degranulation occurs, which causes the release of a wide variety of chemical mediators such as histamine, leukotriene, and prostaglandins [1, 2]. These mediators cause the cardinal symptoms of AR, including sneezing, nasal itching, and nasal congestion [1, 2]. All these reactions occur within minutes after allergen exposure and are referred to as early phase responses [1, 2]. Over 4 to 6 hours after allergen exposure, these mediators, through a complex interplay of several types of events, trigger the recruitment and accumulation of inflammatory cells such as eosinophils, T cells, and mast cells in the nasal mucosa, resulting in the persistent allergic inflammation and the symptoms associated with nasal congestion [1–3]. These responses are referred to as late phase responses and are mainly mediated by eosinophils via the secretion of factors, such as eosinophil cationic protein, major basic protein, and leukotrienes, among others, which cause persistent inflammation and epithelial damage in the nasal mucosa [1, 4]. In addition to these chemical mediators, eosinophils produce free radicals such as O_2_^−^ and H_2_O_2_ [5].

Nitric oxide (NO) was first identified as a vasodilator and is considered a key gaseous signaling molecule that plays a role in a variety of normal physiological processes, following the induction of endothelial nitric oxide synthase (eNOS) and neuronal NOS (nNOS) [6, 7]. On the other hand, the amount of NO produced by inducible NOS (iNOS) from polymorphonuclear leukocytes, such as neutrophils, eosinophils, mast cells, epithelial cells, and fibroblasts after inflammatory stimulation is generally larger than that produced by eNOS and nNOS; moreover, NO causes the oxidation of the components of the cell membrane and tissue injury in the presence of superoxide [7–9]. In patients with AR, some studies have shown increased levels of both iNOS in the nasal mucosa [10] and NO in exhaled air [11–13], suggesting that NO may play an essential role in the development of chronic AR [12, 14].

Quercetin is one of the plant-derived polyphenolic compounds, called flavonoids, which are found in a variety of human diets [15]. Several studies on the beneficial effects of quercetin on human health revealed that it has antimicrobial, anticancer, antidiabetic, and antihypertensive activities [16–18]. Quercetin has also been shown to act as a scavenger of free radicals and to protect the development of oxidative stress responses [19]. In allergic diseases, quercetin has been reported to inhibit the production of inflammatory cytokines, chemokines, and chemical mediators from mast cells and eosinophils* in vitro* [20–23]. Additionally, studies involving experimental murine and rat models of allergy have shown that oral administration of quercetin inhibited the development of allergic symptoms, such as bronchial hyperreactions, scratching, rubbing around the nose, and cyanosis around the tail after a specific allergen challenge [24, 25]. A previous study involving AR model rats showed that quercetin attenuated the clinical symptoms of sneezing and nasal rubbing movements through the suppression of substance P, calcitonin gene-related peptide, and nerve growth factor in the nasal cavity after nasal challenge with toluene diisocyanate [26]. However, the influence of quercetin on NO production has not been sufficiently investigated. Therefore, the present study aimed to assess the influence of quercetin on NO production* in vitro*.

## 2. Materials and Methods

### 2.1. Reagents

Quercetin was purchased from Sigma-Aldrich Co., Ltd. (St. Louis, MO, USA) as a preservative-free pure powder. It was dissolved in dimethyl sulfoxide (DMSO) at a concentration of 10.0 mM and was then diluted with Airway Epithelial Cell Growth Media (AECG medium; PromoCell GmbH, Heidelberg, Germany) at appropriate concentrations for the experiments, sterilized by passing through 0.2 *μ*m filters, and stored at 4°C until use. Recombinant human IL-4 was purchased from R & D Systems, Inc. (Minneapolis, MN, USA), as a preservative-free pure powder. IL-4 was also dissolved in an AECG medium, then sterilized, and stored at 4°C until use. Leflunomide, which is a Janus kinase (JAK)-STAT6 inhibitor [27, 28], was purchased from Sigma-Aldrich Co., Ltd., and was first dissolved in DMSO at a concentration of 1.0 mg/ml. This was followed by dilution in an AECG medium at appropriate concentrations for the experiments. mRNA isolation kits were purchased from Milteny Biotec (Bergisch Gladbach, Germany). The reagents used for the cDNA synthesis and quantitative real-time reverse transcription-polymerase chain reaction (RT-PCR; TaqMan Gene Expression Assays) kit were purchased from Invitrogen Corp. (Carlsbad, CA, USA) and Applied Biosystems (Foster City, CA, USA), respectively.

### 2.2. Cell Culture

Human nasal epithelial cells (HNEpCs), which were established by PromoCell GmbH, were suspended in an AECG medium (PromoCell GmbH) at a concentration of 1 x 10^5^ cells/ml, and were used as a target. To examine the influence of IL-4 on NO production from HNEpCs, 1 x 10^5^ cells (1.0 ml) were introduced in triplicate into 24-well culture plates containing various concentrations (0.5 to 30.0 ng/ml) of IL-4 to make a final volume of 2.0 ml. After 24 to 72 hours, the culture supernatants were collected and stored at -40°C until use. To examine the influence of quercetin and leflunomide on NO production from HNEpCs after IL-4 stimulation, 1 x 10^5^ cells (1.0 ml) were introduced in triplicate into 24-well culture plates containing various concentrations of either quercetin or leflunomide. The cells were then stimulated with 10.0 ng/ml of IL-4 for 48 hours in a solution with a total volume of 2.0 ml. The culture supernatants were collected and stored as mentioned above. To examine the influence of quercetin on the transcription factor, STAT6, activation, and iNOS mRNA expression in HNEpCs, 1 x 10^5^ cells were cultured in a similar manner for 12 and 24 hours, respectively [29]. In all experiments, quercetin and leflunomide were added to cell cultures 2 hours before stimulation.

### 2.3. Assay for NO

The NO levels in the culture supernatants were examined in duplicate using commercially available Griess reagent kits, which can measure NO_2_ and NO_3_ (Dojin Co., Ltd., Kumamoto, Japan), according to the manufacturer's recommended procedures.

### 2.4. Assay for Radical Intensity

The NO-scavenging activity of quercetin was examined using an electron spin resonance (ESR) spectrometer (JOEL JES RE1X, X-band, 100 kHz modulation frequency; JOEL Datum Co., Tokyo, Japan), according to the methods described previously [30]. The radical intensity of NO, which was produced from a reaction mixture of 20 *μ*M carboxy-PTIO and 60 *μ*M NOC-7, was determined in a 0.1 M phosphate buffer (pH 7.4) in the presence of 30% DMSO. Samples were added after 3 min of mixing the reagents. The reaction mixture was then introduced into a capillary (100 x 1.1 mm ID, Drummond Scientific Co., Broomall, PA, USA), which was placed in a quartz cell (270 mm long, 5 mm ID, JOEL Datum Co.). The ESR signal was measured at resonance frequency of 9.4 GHz under the following conditions: center field, 336.0 ± 5.0 mT; microwave power 8 mW; modulation amplitude, 0.1 mT; gain, 250; scan time, 2 min and time constant, 0.03. The radical intensity of NO was defined as the ratio of the signal intensity of the first peak of carboxy-PTIO to that of MnO, and it was expressed relative to the height of MnO, which was an external marker.

### 2.5. Assay for Transcription Factor Activation

STAT6 activity in the cultured cells was examined by measuring the phosphorylated STAT6 level using commercially available ELISA test kits (Abcam plc., Cambridge, MA, USA), according to the manufacturer's recommended procedures.

### 2.6. Qualitative Assay for mRNA Expression

mRNA expression for iNOS in the cultured cells was examined by quantitative real-time RT-PCR. Total mRNA was isolated from the cultured cells using mRNA isolation kit (Milteny Biotec), according to the manufacturer's protocols. First-strand cDNA was synthesized from 2.0 *μ*g of mRNA using a Superscript cDNA synthesis kit (Invitrogen Corp.), according to the manufacturer's instructions with a T100 thermal cycler (Bio-Rad Co., Hercules, CA, USA). The cDNA templates were then amplified by PCR using TaqMan Gene Expression Assays, PCR primers (iNOS; TaqMan Gene Expression Assays; No. Hs01075529_m1 and GAPDH; TaqMan Gene Expression Assays; No. Hs02786624_g1) and RT master mix. Predesigned and validated gene-specific TaqMan Gene Expression Assays [31, 32] were used in duplicate for quantitative real-time RT-PCR, according to the manufacturer's protocols. PCR assays were conducted as follows: denaturation at 95°C for 10 min, 40 cycles of 15 s denaturation at 95°C, and 1 min annealing and extension at 60°C. The samples were analyzed using the ABI Prism 7900HT Fast RT-PCR System (Applied Biosystems) [32]. Relative quantification (RQ) studies [33] were prepared from the data collected [threshold cycle numbers (Ct)] with the ABI Prism 7900HT Sequence-Detection System (SDS) software v. 2.3 (Applied Biosystems).

### 2.7. Statistical Analysis

Significant statistical differences between the control and experimental groups were examined using ANOVA followed by Dunnette's multiple comparison test. Data analysis was performed using ANOVA for Mac (SPSS Inc., Chicago, IL, USA). The level of significance was considered at a P value of < 0.05.

## 3. Results

### 3.1. Influence of Quercetin on NO Production from HNEpCs after IL-4 Stimulation

**Figure 1:**
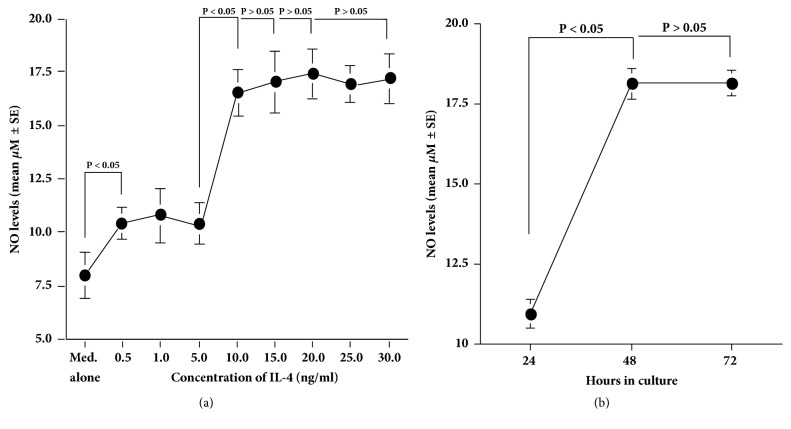
Influence of IL-4 stimulation on NO production from HNEpCs* in vitro*. HNEpCs at 1 x 10^5^ cells/ml are stimulated with different concentrations of IL-4 for 24 to 72 hours. NO levels in the culture supernatants are measured using the Griess method, and the results are expressed as the mean *μ*M ± SE of the triplicate cultures. (a) Dose-response profile of IL-4 on NO production; (b) time course of IL-4-induced NO production. The experiments are performed twice with similar results. Med. alone: medium alone; IL-4: interleukin-4; NO: nitric oxide; HNEpCs: human nasal epithelial cells.

**Figure 2:**
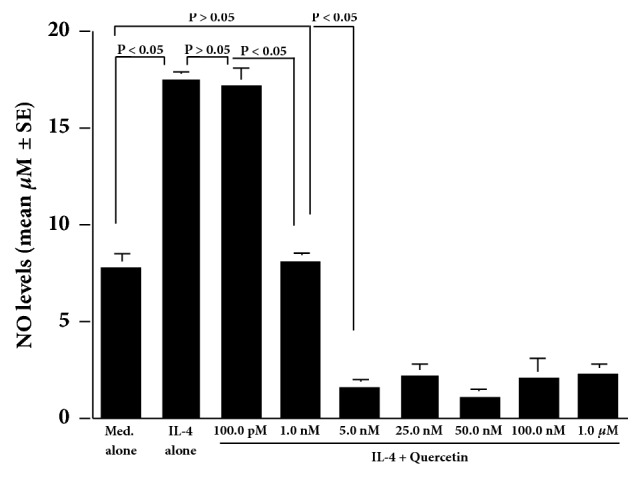
Influence of quercetin on NO production from HNEpCs after IL-4 stimulation* in vitro*. HNEpCs at 1 x 10^5^ cells/ml are cultured with 10.0 ng/ml of IL-4 for 48 hours in the presence and absence of various concentrations of quercetin. The NO levels in the culture supernatants are measured using the Griess method, and the results are expressed as the mean *μ*M ± SE of the triplicate cultures. The experiments are performed twice with similar results. Med. alone: medium alone; IL-4: interleukin-4; NO: nitric oxide; HNEpCs: human nasal epithelial cells.

The first set of experiments showed that IL-4 stimulation caused an increase in NO production from HNEpCs, which was first observed at 0.5 ng/ml and peaked at more than 10.0 ng/ml of IL-4 (Figure 1(a)). The second set of experiments on the time course of NO production from HNEpCs in response to IL-4 stimulation showed that the NO levels peaked at 48 hours and plateaued thereafter (Figure 1(b)). The third set of experiments showed that although treatment of cells with quercetin at 100.0 pM did not inhibit NO production from HNEpCs, quercetin at 1.0 nM significantly suppressed the ability of cells to produce NO in response to IL-4 stimulation. The NO levels in the culture supernatants were nearly identical between the experimental and control groups, but this was not significant (Figure 2). Furthermore, the addition of quercetin at more than 5.0 nM into the cell cultures completely inhibited the ability of the cells to produce NO after IL-4 stimulation. The NO levels in the culture supernatants were extremely lower in the experimental samples than in the control samples (Med. alone; Figure 2).

### 3.2. NO-Scavenging Activity of Quercetin

**Figure 3:**
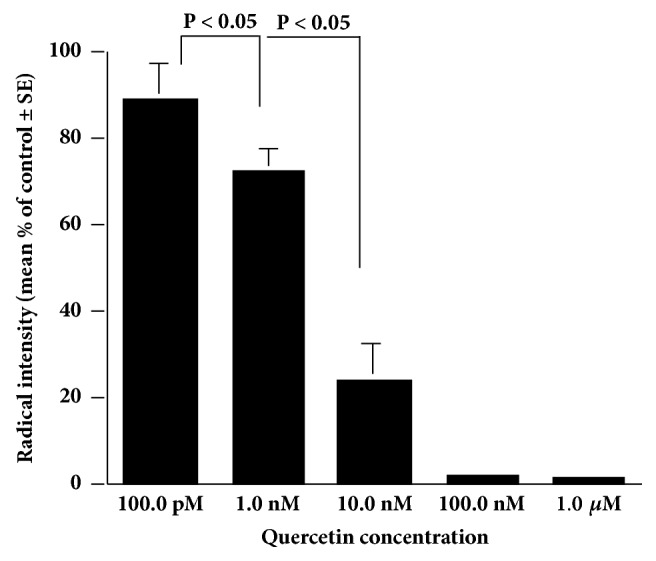
Nitric oxide scavenging activity of quercetin* in vitro*. The reaction mixture of various concentrations of quercetin, NOR-7, and carboxy-PTIO is prepared in phosphate buffer and incubated at 25°C for 30 minutes. Radical intensity is measured with an ESR spectrometer. The experiments are performed twice with similar results.

In ESR study (Figure 3), the NO radical produced by NOC-7 could not scavenged by quercetin at 100.0 pM, was significantly scavenged by quercetin at 1.0 nM, and was completely scavenged by quercetin at more than 100.0 nM.

### 3.3. Influence of Quercetin on STAT6 Activation and iNOS mRNA Expression in HNEpCs after IL-4 Stimulation

**Figure 4:**
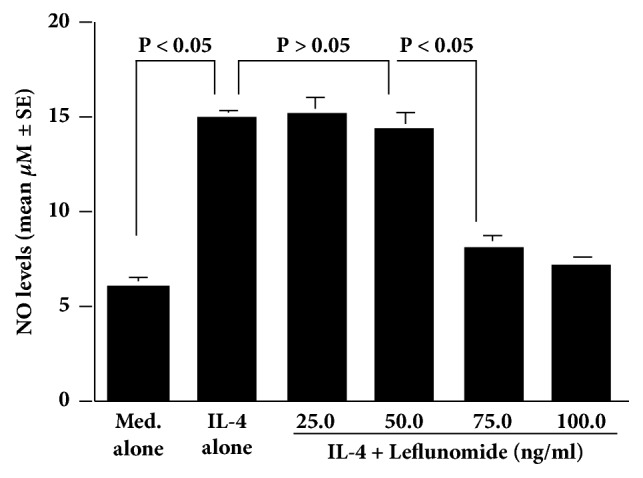
Influence of leflunomide on IL-4-induced NO production from HNEpCs* in vitro. *HNEpCs at 1 x 10^5^ cells/ml are cultured with 10.0 ng/ml of IL-4 for 48 hours in the presence and absence of various concentrations of leflunomide. The NO levels in the culture supernatants are measured using the Griess method, and the results are expressed as the mean *μ*M ± SE of the triplicate cultures. The experiments are performed twice with similar results. Med. alone: medium alone; IL-4: interleukin-4; NO: nitric oxide; HNEpCs: human nasal epithelial cells.

**Figure 5:**
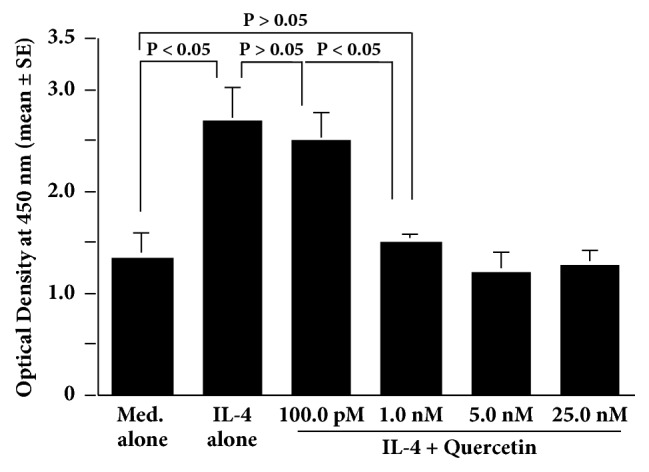
Influence of quercetin on STAT6 activation in HNEpCs after IL-4 stimulation* in vitro*. HNEpCs at 1 x 10^5^ cells/ml are cultured with 10.0 ng/ml of IL-4 for 12 hours in the presence and absence of various concentrations of quercetin. STAT6 activation is measured using ELISA, and the results are expressed as the mean optical density at 450 nm ± SE of the triplicate cultures. The experiments are performed twice with similar results. Med. alone: medium alone; IL-4: interleukin-4; HNEpCs: human nasal epithelial cells.

**Figure 6:**
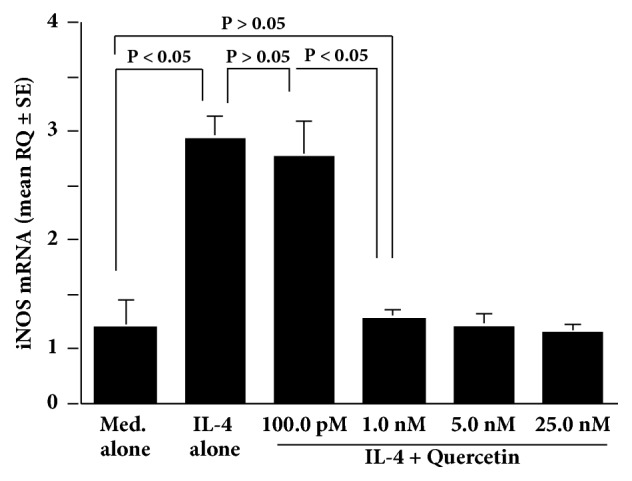
Influence of quercetin on iNOS mRNA expression in HNEpCs after IL-4 stimulation* in vitro*. HNEpCs at 1 x 10^5^ cells/ml are cultured with 10.0 ng/ml of IL-4 for 24 hours in the presence and absence of various concentrations of quercetin. iNOS mRNA expression is measured using quantitative real-time RT-PCR, and the results are expressed as the mean RQ ± SE of the triplicate cultures. The experiments are performed twice with similar results. Med. alone: medium alone; IL-4: interleukin-4; HNEpCs: human nasal epithelial cells; iNOS: inducible nitric oxide synthase; RQ: relative quantification.

The IL-4-induced increase in NO production from HNEpCs was significantly suppressed by leflunomide at more than 75.0 ng/ml but not at less than 50.0 ng/ml (Figure 4). We next examined the influence of quercetin on STAT6 activation in HNEpCs after IL-4 stimulation. Addition of quercetin into the cell cultures at more than 1.0 nM caused significant suppression of the IL-4-induced STAT6 activation (Figure 5). Finally, treatment of cells with quercetin at more than 1.0 nM caused significant suppression of the IL-4-induced iNOS mRNA expression in HNEpCs (Figure 6).

## 4. Discussion

The present study showed that quercetin, at minimum concentration of 1.0 nM, significantly inhibited IL-4-induced NO production from nasal epithelial cells through the suppression of STAT6 activation and iNOS mRNA expression. A previous study reported that, after oral administration of 64 mg quercetin to healthy humans, the plasma levels of quercetin gradually increased, peaked at 650 nM, and decreased to half the peak levels at 24 hours after administration [34]. A dose of 1200 mg quercetin per day is recommended as a dietary supplement that can increase the plasma quercetin concentration to up to 12 *μ*M [34], which is much higher than the level that suppressed NO production* in vitro*. Based on these reports, it is strongly suggested that the results obtained in this study may reflect the* in vivo* effect of quercetin on NO production from nasal epithelial cells after inflammatory stimulation.

Polymorphonuclear leukocytes, including eosinophils and mast cells, are known to play pivotal roles in the development of allergic inflammatory responses through the secretion of inflammatory cytokines and chemical mediators [1–4]. In addition to these mediators, polymorphonuclear leukocytes can produce reactive oxygen species, such as O_2_^−^ and H_2_O_2_, which are responsible for the modification of inflammatory responses [5]. Another reactive oxygen species, which is NO, is also produced by these inflammatory cells [7–9] and plays essential roles as one of the final effector molecules in the pathogenesis of inflammatory responses [12, 14]. NO is generated by several types of mammalian cells and is rapidly oxidized to its more stable metabolites, including nitrite and nitrate. These metabolites react with superoxide to produce the damaging oxidant peroxynitrite, which initiates the oxidation of proteins and lipids in the outer cell membrane, and result in the development of tissue injury [7–9]. The NO that does not completely form peroxynitrite can easily penetrate the cell membrane and react with intracellular superoxide to form peroxynitrite, which causes nuclear membrane and DNA damage and apoptotic cell death in inflammatory tissues [7–9]. To clarify the relationship between the changes in NO and AR development, some researchers reported that the NO levels in the exhaled air were higher in AR patients than in healthy controls [11–14]. NO levels have been found to correlate positively with the computed tomography findings in AR patients [12], and a high nasal NO level might be a useful biomarker of eosinophilic inflammation that is indicative of AR in the nasal mucosa [13, 14]. Along with these findings, the results of the present study indicated that the suppressive effect of quercetin on NO production from nasal epithelial cells might be one of the therapeutic mechanisms of quercetin in AR.

IL-4, which is an immunoregulatory cytokine that is produced predominantly by Th2 type T cells, is well known to play essential roles in the class switching to IgE among B cells and in the maturation of Th2 T cells, which are responsible for the development and persistence of allergic diseases, including AR [35, 36]. IL-4 mediates its functions by interacting with complex receptor systems, including type I and type II receptor systems [36]. IL-4 first attaches to the alpha subunit of the IL-4 receptor, and this complex induces the activation of the tyrosine kinases, JAK 1 and JAK 3, which cause the phosphorylation of the transcription factor, STAT6 [36, 37]. Phosphorylated STAT6 then migrates to the nucleus and binds to the promoter regions of IL-4 responsive genes, resulting in the mRNA expression that is essential for the production of inflammatory mediators, including NO [34]. Therefore, the second part of the experiments was undertaken to examine whether quercetin could inhibit NO production from nasal epithelial cells after IL-4 stimulation through the suppression of this signaling pathway. The present results showed that treatment of nasal epithelial cells with leflunomide at more than 75.0 ng/ml caused significant inhibition of IL-4-induced NO production. Leflunomide was reported to inhibit the tyrosine phosphorylation of both JAK3 and STAT6 [27, 28]. Activation of JAK3 was also reported to correlate with the tyrosine phosphorylation of STAT6 [27]. From these reports, the present results strongly suggested that the JAK/STAT signaling pathway is essential for NO production from nasal epithelial cells after IL-4 stimulation and that quercetin inhibits IL-4-induced NO production from nasal epithelial cells through the suppression of this signaling pathway, especially STAT6 activation. This speculation might be supported in part by the present findings that IL-4-induced STAT6 activation and iNOS mRNA expression were significantly suppressed by treatment of nasal epithelial cells with quercetin at more than 1.0 nM. There is also another possibility that quercetin inhibits the phosphorylation of JAK3, followed by suppression of STAT6 activation and iNOS mRNA expression, resulting in inhibition of NO production. Further experiments are required to clarify this point.

JAK3 and STAT6 phosphorylation require an increase in intracellular Ca^2+^ levels [38, 39]. A previous study reported that quercetin can inhibit the increase in intracellular free Ca^2+^ levels in human mast cells after inflammatory stimulation* in vitro* [40]. Quercetin has also been reported to inhibit the phosphorylation of several types of tyrosine kinases that are responsible for transcription factor activation [41, 42]. Based these results, quercetin might inhibit the phosphorylation of tyrosine kinases by inhibiting an increase in Ca^2+^ levels in nasal epithelial cells after IL-4 stimulation, resulting in suppression of both iNOS production and NO generation. Further experiments are required to clarify this assumption.

## 5. Conclusion

Quercetin exerted inhibitory effects on IL-4-induced NO production in HNEpCs. These findings suggested that the ability of quercetin to suppress NO production from nasal epithelial cells may account, at least in part, for the clinical efficacy of quercetin in AR.

## Data Availability

The data used to support the findings of this study are available from the corresponding author upon request.
